# Green Extraction of Antioxidant Flavonoids from Pigeon Pea (*Cajanus cajan* (L.) Millsp.) Seeds and Its Antioxidant Potentials Using Ultrasound-Assisted Methodology

**DOI:** 10.3390/molecules26247557

**Published:** 2021-12-13

**Authors:** Duangjai Tungmunnithum, Samantha Drouet, Jose Manuel Lorenzo, Christophe Hano

**Affiliations:** 1 Department of Pharmaceutical Botany, Faculty of Pharmacy, Mahidol University, Bangkok 10400, Thailand; 2 Laboratoire de Biologie des Ligneux et des Grandes Cultures, INRAE USC1328, Campus Eure et Loir, Orleans University, 28000 Chartres, France; samantha.drouet@univ-orleans.fr; 3 Le Studium Institue for Advanced Studies, 1 Rue Dupanloup, 45000 Orléans, France; 4 Centro Tecnológico de la Carne de Galicia, Adva. Galicia n° 4, Parque Tecnológico de Galicia, San Cibrao das Viñas, 32900 Ourense, Spain; jmlorenzo@ceteca.net; 5 Área de Tecnología de los Alimentos, Facultad de Ciencias de Ourense, Universidad de Vigo, 32004 Ourense, Spain

**Keywords:** green extraction, antioxidant flavonoids, pigeon pea, *Cajanus cajan* (L.) Millsp, ultrasound

## Abstract

Pigeon pea is an important pea species in the Fabaceae family that has long been used for food, cosmetic, and other phytopharmaceutical applications. Its seed is reported as a rich source of antioxidants and anti-inflammatory flavonoids, especially isoflavones, i.e., cajanin, cajanol, daidzein, and genistein. In today’s era of green chemistry and green cosmetic development, the development and optimization of extraction techniques is increasing employed by the industrial sectors to provide environmentally friendly products for their customers. Surprisingly, there is no research report on improving the extraction of these isoflavonoids from pigeon pea seeds. In this present study, ultrasound-assisted extraction (USAE) methodology, which is a green extraction that provides a shorter extraction time and consumes less solvent, was optimized and compared with the conventional methods. The multivariate strategy, the Behnken–Box design (BBD) combined with response surface methodology, was employed to determine the best extraction conditions for this USAE utilizing ethanol as green solvent. Not only in vitro but also cellular antioxidant activities were evaluated using different assays and approaches. The results indicated that USAE provided a substantial gain of *ca* 70% in the (iso)flavonoids extracted and the biological antioxidant activities were preserved, compared to the conventional method. The best extraction conditions were 39.19 min with a frequency of 29.96 kHz and 63.81% (*v*/*v*) aqueous ethanol. Both the antioxidant and anti-aging potentials of the extract were obtained under optimal USAE at a cellular level using yeast as a model, resulting in lower levels of malondialdehyde. These results demonstrated that the extract can act as an effective activator of the cell longevity protein (SIR2/SIRT1) and cell membrane protector against oxidative stress. This finding supports the potential of pigeon pea seeds and USAE methodology to gain potential antioxidant and anti-aging (iso)flavonoids-rich sources for the cosmetic and phytopharmaceutical sectors.

## 1. Introduction

*Cajanus cajan* (L.) Millsp is an edible pea species of the family Fabaceae that has long been used as an ingredient for food, in cosmetics, and as medicines since ancient times, especially in Asian, Egyptian, and African counties [1,2,3,4,5,6,7]. This plant species is well-known as its common name, pigeon pea [1,7,8]. In addition, local people in different regions use different vernacular names to identify *C. cajan*, i.e., Thaua Maha (ถั่วมะแฮ), Thaua Hae (ถั่วแฮ), Thuareaphi (ถั่วแระผี), Thuamaetay (ถั่วแม่ตาย), and Phanosae (พะหน่อเซะ). *C. cajan* is distributed in Asia and Africa and is currently introduced to the United States of America as well as some parts of Europe, for example, Switzerland [1,8,9]. However, the largest pigeon pea producer is the Asian region due to its suitable environment.

**Figure 1:**
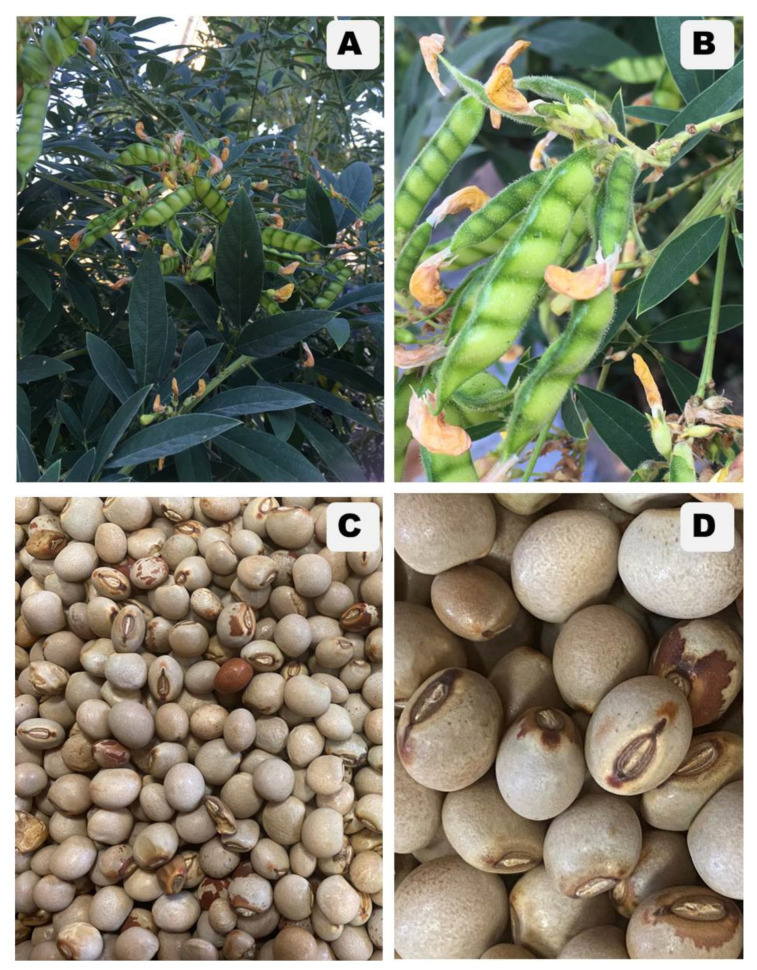
*Cajanus cajan* (L.) Millsp: (**A**). Habitat; (**B**). Inflorescence and fruits; (**C**,**D**) Mature and dried seed. The photos were taken by D.T. and C.H. on 25 October 2021.

This edible pea species is a terrestrial and perennial shrub, with an erect stem that is 100–350 cm tall, and branchlets that are pubescent green to gray. Its leaves are stipulate, pinnately 3-foliolate with an ovate to lanceolate shape, apex acute or acuminate; and petiole 1–5 cm long. The abaxial of the leaf is densely pubescent, consisting of inconspicuous yellow glands. The adaxial of the leaf is pubescent. Inflorescence is racemose type with ovate or ovate-elliptic bracts, 3.5–8.0 cm long, peduncle 1.5–4.0 cm. Each flower consists of green, pubescent, campanulate calyx, yellow, and papilionaceous form of corolla: suborbicular with auricle standard, obovate with auricle wings, and obtuse apex keel. Its ovary is pubescent with a slender and glabrous style and capitate stigma, and numerous ovules. Its fruit is an oblong or linear-oblong legume, subspherical, and gray with brown spotty seeds [1,10] (Figure 1). The flowering season of pigeon pea ranges between June and November each year, although sometimes flowering occurs until January [1,3,10]. This pea species is normally reproduced by seeding that falls down into in the field during the rainy season.

Generally, the seed is the most valuable used part of this plant species, where the young seed is consumed as a side dish as a spicy salad or soup. It can be also used as an important ingredient in many types of curry. Besides being a protein-rich food source, *C. cajan* seed is used as a traditional medicine, i.e., local Indian people use this plant for the treatment of stomatitis and gingivitis [11]. Furthermore, local people in Oman have used the seed of this plant to cure various kinds of chronic diseases [12]. In addition, it is also suggested as a raw material for cosmetics and skincare due to several reports on its flavonoids and phenolic acids, as well as its antioxidant and anti-inflammatory activities [1,2,4,13,14,15,16,17].

Pigeon pea is a rich source of various phytochemicals including flavonoids, stilbenes, and coumarins [1]. Flavonoids are C6-C3-C6 backbone phenylpropanoids with two phenyl rings (rings A and B) and one heterocyclic ring (ring C) (Figure S1). Flavonoids can be split into several subgroups based on the carbon of the C ring linked to the B ring, as well as the degree of unsaturation and oxidation of the C ring. Isoflavones are flavonoids with the B ring linked to position 3 of the C ring [18] (Figure S1). Pigeon pea seeds (beans) are particularly rich in flavonoids, mostly in isoflavones, such as cajanin, cajanol, daidzein, and genistein [12,13,18,19]. Several biological activities of pigeon pea extracts, including antioxidant and anti-inflammatory activities, have been identified in the recent decade, with potential application in cosmetics [14,18,20]. The development and optimization of extraction techniques are crucial for future industrial applications. To date, however, no study on improving the extraction of (iso)flavonoids from pigeon pea seeds has been reported, thus severely restricting the potential use of this valuable resource. For cosmetic applications, green extraction processes must be considered. Microwave-assisted extraction [21,22], pressured liquid extraction [23], and enzyme-assisted [24] or ultrasound-assisted extraction (USAE) [25,26,27,28,29,30] are only a few of the green extraction strategies for plant natural products that have been published so far. In the present work, we concentrated on USAE, which is one of the most basic and cost-effective strategies for increasing the plant extraction yield, in particular for (iso)flavonoids extraction [30,31,32,33]. When compared to other traditional extraction procedures, USAE presents a shorter extraction time and generally uses less solvent, making it a green extraction approach that can be quickly scaled up for commercial application [34,35]. In particular, the benefits of USAE are based on greater mass transfer and cell disruption operating under mid-extraction conditions, as well as better solvent cell penetration, which improves the extraction and capillary effects and minimizes phytochemical degradation [34,35,36].

A multivariate strategy (Behnken–Box design) combined with response surface methodology was used to determine the best extraction conditions for this USAE utilizing ethanol as a green solvent. Both in vitro cell-free and cellular antioxidant activity were assessed using different assays and approaches. The results were compared to those obtained using standard heat reflux extraction (HRE) and a reference antioxidant. The flavonoid-rich extract from pigeon pea seeds was subjected to HPLC-UV-DAD analysis.

## 2. Results

### 2.1. Extraction Optimization Using the Box–Behnken Design (BBD)

The extraction of flavonoids from pigeon pea seeds by ultrasound was optimized using a Box–Behnken design (BBD). Table 1 shows the five parameters that were properly considered (i.e., ultrasound frequency, extraction duration, aqueous ethanol (EtOH) concentration, extraction temperature, and liquid/solid ratio) as well as their levels.

Table 2 shows the experimental (mean and standard error of three independent experiments) and predicted total flavonoid contents (TFCs) obtained after USAE from pigeon pea seeds for the 41 different observations (run ID) corresponding to the different USAE conditions of the Behnken–Box matrix that were randomly determined (run order) generated by the XL-Stat software.

**Figure 2:**
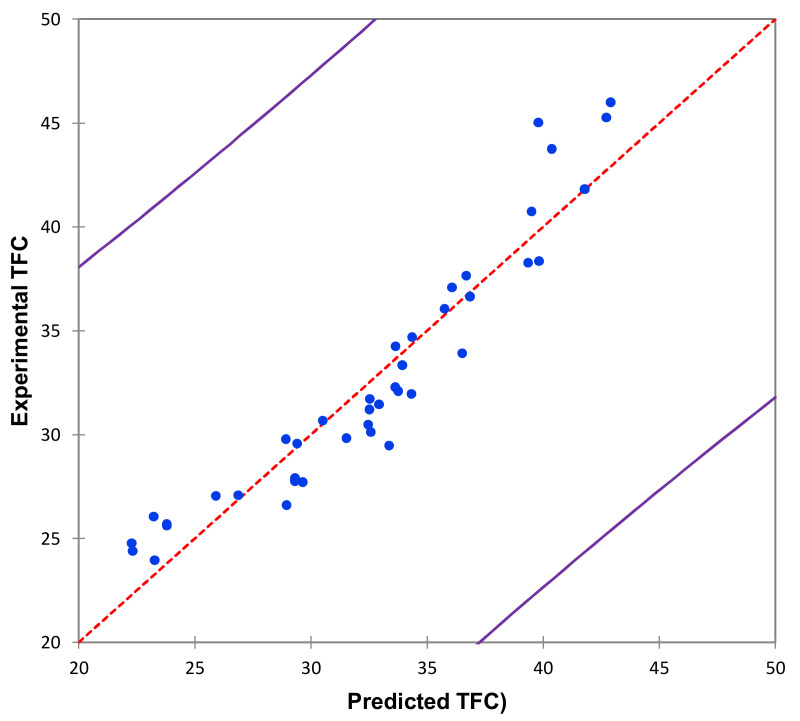
Model-predicted TFC as a function of the experimentally measured TFC (expressed in mg/100 g DM).

Using these extraction conditions, TFC varied from 23.94 (Obs1, Table 2) to 45.98 (Obs38, Table 2) mg total flavonoid per 100 g of pigeon pea seed dry materials. The experimental and predicted values have a high degree of correlation and no statistically significant difference (*p* < 0.05) was observed between experimental and predicted values, evidencing the model’s robustness (Figure 2).

Table 3 shows the results of the analysis of variance (ANOVA) and the model’s fit. The high F-value (19.516) and low *p*-value (*p* < 0.0001) confirmed that the model is highly significant. The determination coefficient (R^2^ = 0.929) and its adjusted value (adjusted R^2^ = 0.881) both demonstrated that this model is adequate for predicting TFC extracted from pigeon pea seeds. The variation coefficient value (CV = 0.715%) also supported the model validity and experimental values.

The results of TFC (Y_TFC_, expressed in mg/g DM) as a function of the five independent extraction factors were subjected to multiple regression analysis to generate the extraction model described by the following polynomial Equation (1):

Y_TFC_ = 41.797 − 0.502X_1_ + 4.119X_2_ + 3.062X_3_ + 0.558X_4_ − 0.705X_5_ − 7.751X_1_^2^ − 6.671X_2_^2^ − 7.938X_3_^2^ + 0.159X_4_^2^ − 1.303X_5_^2^ − 0.478X_1_X_2_ + 0.260X_1_X_3_ − 0.498X_1_X_4_ + 0.001X_1_X_5 +_ 2.325X_2_X_3_ + 2.763X_2_X_4_ + 2.124X_2_X_5_ + 2.190X_3_X_4_ − 0.539X_3_X_5_ − 0.998X_4_X_5_ (1)

The linear coefficients X_2 and_ X_3_; the quadratic coefficients X_1_^2^, X_2_^2^, and X_3_^2^; and the interaction coefficient X_2_X_3_ and X_2_X_4_ proved to have a significant influence on the TFC recovered from pigeon pea seeds, according to the statistical analysis (Table 4). The remaining linear, quadratic, and interaction coefficients, on the other hand, were not statistically significant at *p* > 0.05. As a result, ultrasonic frequency (X_2_), ethanol concentration (X_3_), and their interaction (X_2_X_3,_ ultrasonic frequency × ethanol concentration), as well as duration (X_1_^2^) and the interaction between ultrasonic frequency and extraction temperature (X_2_X_4_, ultrasonic frequency × extraction temperature), appeared to have a significant impact on the extraction efficiency of TFC from pigeon pea seeds. A positive coefficient value indicates that increasing the level of this parameter had a positive overall influence on the extraction yield, whereas a negative value indicates that the extraction yield decreased as the level of the considered parameter was increased. Here, the linear and interaction coefficients were all positive, whereas the quadratic coefficient was negative, indicating a complex effect of all these parameters on the extraction efficiency.

**Figure 3:**
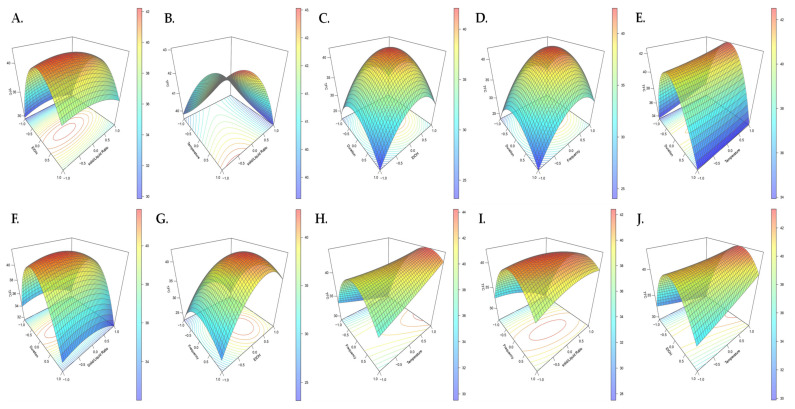
Surface response plots of the TFC extracted from pigeon pea seeds (in mg/g DM) as a function of (**A**) the EtOH concentration and solid/liquid ratio; (**B**) temperature and solid/liquid ratio; (**C**) duration and EtOH concentration; (**D**) duration and extraction frequency; (**E**) duration and temperature; (**F**) duration and solid/liquid ratio; (**G**) US frequency and EtOH concentration; (**H**) US frequency and temperature; (**I**) US frequency and solid/liquid ratio; (**J**) EtOH and temperature.

The complexity of the USAE of TFC from pigeon pea seeds was addressed by using response surface plots (Figure 3). The 3D plots that resulted depicted the positive effects on TFC caused by increasing the ultrasonic frequency in combination with increasing the ethanol concentration (X_2_X_3_) or extraction temperature (X_2_X_4_) (Figure 3G,H), which were in good agreement with the positive values of the corresponding linear and interaction coefficients determined for these parameters (Table 4). The TFC, on the other hand, passed through a maximum, illustrating that the quadratic coefficients X_1_^2^, X_2_^2^, and X_3_^2^ were negative. In this study, TFC was reduced when a high ultrasound frequency was combined with a prolonged extraction time or temperature, as well as a high ethanol concentration. This decrease could be the result of partial destruction or reduced solubilization of some less stable flavonoids [35].

According to the adjusted second-order polynomial equation, the best extraction conditions were 39.19 min in an ultrasonic bath with a frequency of 29.96 kHz (here adjusted to 30 kHz taking into account the US device’s limitations) and 63.81 percent (*v/v*) aqueous ethanol used as a solvent. The temperature was set at 45 °C, and the solid to liquid ratio was set to 5.5 mg/mL solvent. Under these conditions, 48.96 ± 0.54 mg/100 g DM TFC were extracted from pigeon pea seeds.

### 2.2. Characterization of Antioxidant and Anti-Aging Potential

#### 2.2.1. Correlation between TFC and In Vitro Antioxidant Capacity

**Figure 4:**
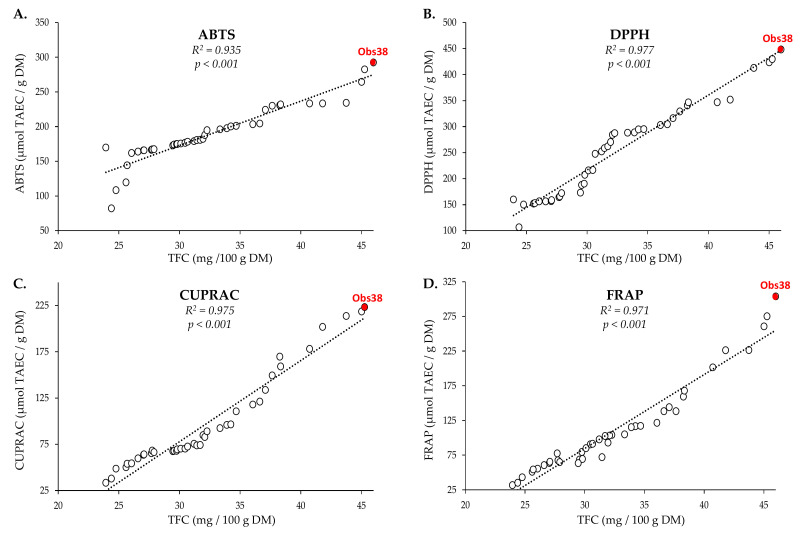
Relation between the TPC in the 41 extracts generated during the BBD optimization and their respective antioxidant capacity determined using the in vitro assays: (**A**) ABTS, (**B**) DPPH, (**C**) CUPRAC, and (**D**) FRAP. Correlation coefficients (R^2^) and *p* values calculated using PAST3.0 are also provided. Actual values for each assay are provided in Table S1 (Supplementary Materials). TEAC: Trolox equivalent antioxidant capacity; TFC: total flavonoid content; DM: dry matter.

To ensure that the potential biological activities were preserved during the extraction process, the antioxidant capacity of the 41 extracts generated during the BBD optimization was determined using four different types of in vitro cell free assays, each with different chemistry of the antioxidant reaction and different mechanisms (Figure 4; Table S1).

These in vitro cell-free antioxidant assays can be classified into different categories based on the chemical reaction involved, with an ABTS assay based on a hydrogen atom transfer reaction (HAT), FRAP and CUPRAC assays based on an electron transfer reaction (ET), and the DPPH assay as a mixed assay [37,38]. The results of these tests were expressed in µmol of Trolox equivalent antioxidant capacity (TEAC) per gram of dry matter (DM) to facilitate comparisons (Table S1). For each assay, there is a strong and highly significant correlation between TFC and the antioxidant capacity, with the highest antioxidant capacity measured for sample Obs38, the closest to optimal extraction conditions. This shows that the biological antioxidant activity is retained throughout extraction and that the resultant extract can be employed for a variety of applications.

#### 2.2.2. Cellular Antioxidant and Anti-Aging Potential

**Figure 5:**
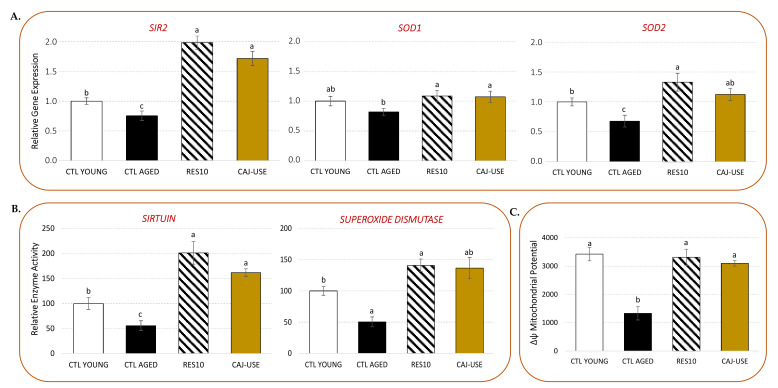
Effects of pigeon pea seed extract obtained under optimal USAE (CAJ-USE) on: (**A**) *SIR2*, *SOD1*, and *SOD2* gene expression determined by RT-qPCR (expression normalized with the alpha-tubulin *TUB1* gene and expressed relative to young yeast cells (CTL YOUNG); (**B**) Sirtuin (SIRT) and total SOD enzyme activities; (**C**) the mitochondrial potential (Δψm) variation used to estimate mitochondria integrity. CTL YOUNG are young yeast cells (day 2 of cultivation). CTL AGED are aged yeast cells (day 5 of cultivation). RES: E-resveratrol, 10 µM (positive control drug). CAJ-USE: pea seed extract obtained under optimal USAE, 1 mg/mL. Values are means ± standard deviations (SD) of 6 independent experiments. Different letters represent significant differences (*p* < 0.05).

The antioxidant and anti-aging potential of the pigeon pea seed extract obtained under optimal USAE (CAJ-USE) was explored at a cellular level using yeast as a model. When compared to young yeast, aged yeast had significantly lower expression of the antioxidant and anti-aging *SIR2* (*silent information regulator 2*), *SOD1* (superoxide dismutase 1, cytoplasmic), and *SOD2* (superoxide dismutase 1, mitochondrial) genes (Figure 5A). The positive control drug, E-resveratrol (RES at 10µM), can reverse this tendency.

In the presence of CAJ-USE (1 mg/mL), *SIR2* gene expression was significantly increased, which was comparable to the observed RES-induced stimulation (Figure 5A). On the contrary, compared to the CTL YOUNG cells, CAJ-USE primarily acts to maintain *SODs* gene expression, which showed only a minor increase: slight for *SOD1* and a little more pronounced for *SOD2*. This trend, however, is comparable to those of RES. However, when compared to the CTL AGED cells, this represents a significant gene expression increase for both *SODs* genes (Figure 5A).

The impact of these gene expression activations was then studied at the enzyme level (Figure 5B). CTL AGED cells had low SIRT and SOD enzyme activities, whereas CTL YOUNG and RES-treated yeast had significantly higher SIRT and SOD activities. CAJ-USE application resulted in significant stimulation of SIRT activity, comparable to that of RES, which was consistent with the gene expression data. Surprisingly, a similar activation profile was observed with total SOD activity. Note that total SOD activity results from the action of both cytosolic and mitochondrial SOD activities. So, this discrepancy could be explained by the fact that this enzyme transcriptional level is not rate limiting, or by the fact that the reference gene (alpha-tubulin) is inadequate for monitoring gene expression of both nuclear and mitochondrial genes. It is worth noting that the observed increase in total SOD activity matched the mitochondrial *SOD2* gene expression better than the cytosolic *SOD1* gene expression. Moreover, CAJ-USE, like RES, was discovered to be capable of maintaining a functional mitochondrial potential Δψm value at the same level as for CTL YOUNG cells (Figure 5C).

**Figure 6:**
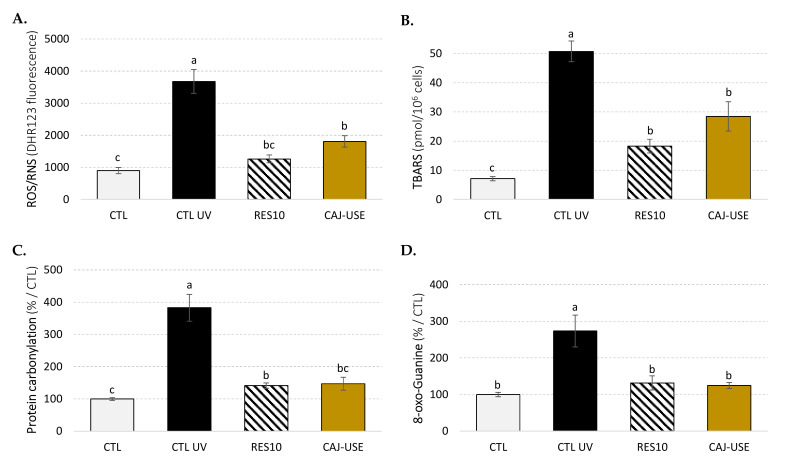
Impact of UV-C-induced oxidative stress on DBY746 yeast on: (**A**) reactive oxygen and nitrogen species (ROS/RNS) production was evaluated with the dihydrorhodamine 123 (DHR123) probe; (**B**) malondialdehyde (MDA) levels measured by TBARS assay; (**C**) protein carbonylation; (**D**) 8-oxo-Guanine formation. CTL: control cells. RES: E-resveratrol, 10 µM (positive control drug). CAJ-USE: pea seed extract obtained under optimal USAE, 1 mg/mL. Values are means ± standard deviations (SD) of 6 independent experiments. Different letters represent significant differences (*p* < 0.05).

These results suggest that the CAJ-USE anti-aging properties may be related to its antioxidant activity. The impact of UV-C-induced oxidative stress on DBY746 yeast was next evaluated (Figure 6).

CAJ-USE supplementation’s significant improvement of yeast survival (Figure S2) subjected to UV-C-induced oxidative stress is related to its ability to significantly lower ROS/RNS formation (Figure 6A) under these conditions. As a result, the impact of oxidative stress was mitigated: CAJ-USE-supplemented yeast cells had lower levels of MDA, indicating that this extract can act as an effective cell membrane protector against oxidative stress, but also lower levels of protein carbonylation and 8-oxo-guanine formation.

### 2.3. HPLC-UV-DAD Analysis and Comparison with the Conventional Method

#### 2.3.1. HPLC-UV-DAD Analysis

**Figure 7:**
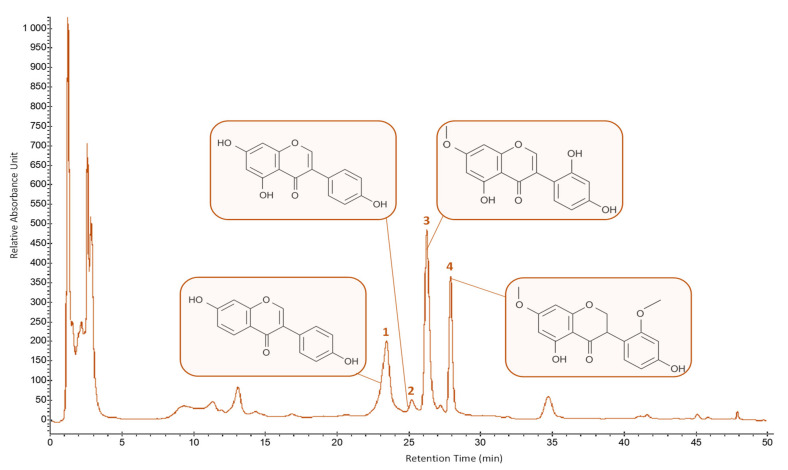
HPLC-UV-DAD chromatogram (recorded at 260 nm) of extract from pigeon pea seeds obtained using optimal USAE conditions. 1: daidzein; 2: genistein; 3: cajanin; 4: cajanol.

The separation of the four key isoflavonoids from pigeon pea seeds: daidzein (1), genistein (2), cajanin (3), and cajanol (4), obtained employing optimal USAE conditions, was done by HPLC-UV-DAD chromatography (here recorded at 260 nm) using the conditions developed for isoflavonoids separation [39] (Figure 7).

Cajanin (18.11 ± 0.27 mg/100 g DM) is the main isoflavonoid from our extract followed by cajanol (11.64 ± 0.17 mg/100 g DM), daidzein (9.03 ± 0.14 mg/100 g DM), and genistein (0.78 ± 0.02 mg/100 g DM).

#### 2.3.2. Comparison with the Conventional Extraction Method

The efficiency of the current USAE method was compared to that of conventional heat reflux extraction (HRE) under the same conditions, except for the US application: EtOH 63.81% (*v/v*) as a solvent for 39.19 min in a water bath at 45 °C and a solid to liquid ratio of 5.5 mg/mL. Table 5 shows the comparison in terms of TFC, the content of the four main isoflavonoids, and the in vitro antioxidant activity. The DPPH test was chosen as the in vitro antioxidant assay for comparison since it had the highest correlation and significance levels (see Section 2.2.1).

When comparing the optimized USAE (48.96 0.54 mg/100 g DM) to the conventional HRE (28.77 4.01 mg/100 g DM), a substantial gain of 70% in (iso)flavonoids extracted from pigeon pea seeds was reported (Table 5). Similarly, the in vitro antioxidant activity follows the same trend, demonstrating the value of the present method for future applications.

## 3. Discussion

Many factors can affect the extraction of phenolic compounds from complex plant matrices [35,40], but three of them stand out when establishing a USAE method: the solvent, the ultrasound frequency used, and the extraction duration [35,41,42].

When establishing an extraction process, the choice of the solvent is a critical parameter to define, and it is determined by the type of application planned. The polarity of the solvents employed for (iso)flavonoid extraction is usually taken into consideration [43]. Flavonoids are commonly extracted from plant material using an alcohol, such as EtOH, water, or a mixture of the two [30,33,35,41,44,45,46]. It is worth noting that the concentration of aqEtOH for optimal outcomes is also dependent on the plant matrix [35,36,44]. EtOH was also considered as an extraction solvent in this study because our objective was to design an extraction process that followed the green chemistry principles for potential nutraceutical and/or cosmeceutical applications of the resulting extract. Indeed, this solvent adheres well to the triple bottom line accounting system, which consists of three parts: social (people), environmental (planet), and financial (money or profit). For instance, EtOH is a less harmful solvent for people, more environmentally friendly than other organic solvents such as methanol, and quite inexpensive [47]. Its extraction capacity may also be easily modulated by adding water, making it a suitable solvent for the extraction of a wide spectrum of polyphenols with varied polarity [48]. Finally, these two universal solvents (EtOH and water) are already widely utilized for applications in food, nutraceuticals, and cosmeceuticals [30,31,32,33,41,47].

Because of its significant impact on extraction efficiency, the US frequency is an important variable to consider. The US frequency has a considerable impact on the cavitation effect and the diffusion coefficient of the chemicals in the extraction solvent [35,36,44]. The US frequency leads to shortening of the extraction duration by acting on the cavitation effect and diffusion coefficient [35,36,44]. As a consequence, the US frequency may enhance the extraction yield by increasing the solubilization of the molecule in the extracting solvent [35,36,44]. However, depending on the molecule and the plant matrix treated to the extraction, a high US frequency might modify the compound’s native/natural structure, reducing not only the extraction yield but also its biological activity, therefore negating any value interest [35,36,44]. As a result, while developing a USAE process, the US frequency must be carefully tuned based on the plant matrix and the phytochemical(s) to be extracted, and the maintenance of the biological activity has to be checked.

The extraction time is also a crucial parameter to consider, especially during USAE, because an increase in the duration does not always imply an increase in the extraction yield. In fact, in the case of USAE, a longer extraction time may often result in increased degradation of the bioactive chemicals [41,49]. As observed in the present study, extending the extraction time during USAE, especially in the presence of water, has been found to cause polyphenol oxidation, lowering the antioxidant potential of the resultant extract dramatically [41,46]. As a result, all of these extraction parameters should be precisely optimized, with any potential interactions taken into account, to avoid not only a significant decrease in extraction yield (both quantitatively and qualitatively), but also a significant decrease in the biological activity of the sample extract. This is why a multivariate methodology was used to optimize flavonoids extraction from pigeon pea seed in this study. Multivariate techniques are particularly successful in optimizing the extraction procedure from complex plant matrices, such as food items and by-products [50]. The Behnken–Box design is one of the most successful multivariate techniques [50,51].

According to the principles of green chemistry [52], the USAE approach described in this work appears to be of great interest, not only in terms of efficiency and use of a renewable green solvent, but also in terms of minimizing energy usage. A reduced extraction time diminish the environmental effect in terms of energy use [53]. Here, compared with the conventional HRE, a substantial gain of 70% in (iso)flavonoids extracted from pigeon pea seeds was reported, while the biological antioxidant activity was preserved. As a result, the USAE procedure established in this study appears to be of significant relevance in terms of green chemistry principles [52], not only in terms of using a renewable green solvent, but also in terms of minimizing energy usage. This efficiency could be explained in part by the hot spot hypothesis, which states that after cavitation bubbles collapse, they act locally as a microreactor, generating a high-temperature and -pressure environment in the surrounding solvent, resulting in more efficient rupture of the plant matrix subjected to extraction and increased release as well as solubilization of flavonoids [33,47].

The concentrations that were reported for TFC, daidzein, and genistein in this study were within the range of previous values for TFC, as well as levels [12,13,18,19]. The observed small variations can be attributed to a variety of genetic backgrounds, as well as environmental factors, such as location (i.e., soil conditions) and climate, which have been shown to have a significant impact on the accumulation of phenolic compounds [47,54]. The current work is the first attempt to provide a relative quantification of cajanin and cajanol contents in pigeon pea seeds.

Due to the complex nature of phytochemicals, and in particular, since the determination of antioxidant activity is highly dependent on the reaction mechanism involved, the antioxidant activity of plant extracts cannot be measured by a single approach. To quantify the antioxidant activity and define the antioxidant mechanism of action of a plant extract, several chemical or biological experiments are required [38]. In vitro cell-free antioxidant tests based on several modes of reaction may give insight into the chemistry behind the antioxidant activity of a plant extract. ABTS and FRAP are based on hydrogen atom transfer reactions (HAT) and electron transfer reactions (ET), respectively, whereas DPPH is a mixed HAT- and ET-based assay [38,55,56].

Although these in vitro studies are interesting from a strictly predictive view based on chemical reactions, they might not always reflect in vivo systems. The validity of these antioxidant studies must thus be viewed as confined to a chemical reactivity interpretation about the considered radicals produced in vitro and must be validated in vivo. The antioxidant activity of these nine extracts has also been researched further for their potential to prevent the lipid peroxidation membrane formed by oxidative stress induced by UV-C in yeast cells, to obtain a better knowledge and better reflect the in vivo scenario. In the context of cellular oxidative stress, yeast cells are an effective model for measuring the antioxidant capability in vivo [57]. Yeast cells have been considered as a good model for assessing the antioxidant capability in response to cellular oxidative stress in vivo [57,58]. It is an appealing and dependable eukaryotic model with well-known mechanisms of defense and adaptability to oxidative stress that may be extended to human cells with more complicated but well-conserved systems [57,58].

The present results support the trend found utilizing in vitro cell-free antioxidant tests at a cellular level, confirming the current extraction method’s potential for creating effective antioxidant extracts from pigeon pea seeds. Our results also show that the extract’s antioxidant action is linked to its flavonoid concentration. Flavonoids, which are powerful natural antioxidants found in food, may be able to mitigate the harmful effects of excessive ROS and RNS production in cells [18,59]. Our extract was able to reduce the harmful effects of ROS/RNS on numerous cellular components, such as membrane lipids, proteins, and DNA. This effect might be connected to mitochondrial preservation during aging and in response to UV-C-induced oxidative stress. Mitochondria generate ROS and RNS as by-products of cellular metabolism in a normal and continuous manner. As a direct result of redox cellular imbalances, the generation of ROS and RNS rises with age, stress, or pollution, potentially leading to the development of numerous degenerative diseases [60,61]. Both SIR2 and SODs have been linked to the activity of our pigeon pea seed extract (CAJ-USE) at both gene expression and enzyme levels. SIR2 is a nicotinamide adenine dinucleotide-dependent protein deacetylase that has been connected to the oxidative stress response, namely the ROS-driven mitochondrial-mediated response [61]. SOD2 is a mitochondrial Mn-SOD that plays an important role in the antioxidant response by effectively scavenging ROS [62]. In several models, SIR2 (also known as SIRT1) has been postulated as an inducer of SOD2 gene expression [63,64]. In line with our results, the stimulation of SIR2 and SOD2 gene expression by various plant-derived natural compounds has been linked to an improved antioxidant capacity [65,66]. Therefore, our extract shows the activation capacity of cell longevity protein (SIR2/SIRT1), as resveratrol is already used in cosmetics as a bioactive ingredient [67].

For cosmetic applications, natural antioxidants have piqued interest in the last decade not only as a bioactive as a potential replacement for possibly harmful synthetic antioxidants like butylated hydroxyanisole (BHA) and butylated hydroxytoluene (BHT) in various food and cosmetic formulations [55,56,68]. Some natural antioxidant phenolics have already been proven to be equally effective as synthetic antioxidants in stabilizing nonpolar systems like bulk oil or other forms of emulsions [55,56]. Therefore, the present study suggests that pigeon pea extracts produced using the current optimized USAE might be used as natural antioxidants in cosmetics.

## 4. Materials and Methods

### 4.1. Chemicals and Reagents

The extraction solvents utilized in this study (ethanol and water) were of analytical grade (Thermo Scientific, Illkirch, France). Merck (Saint-Quentin Fallavier, France) was the provider of the reagents for the antioxidant assays and standards.

### 4.2. Plant Materials

Pigeon pea seeds were purchased from a local market imported from UK (TRS Wholesale Co., London, UK). Seeds were ground using a professional grinder (Grindomix GM200, Retsch, Eragny, France) at maximum speed (10,000 rpm) for 30 s.

### 4.3. Ultrasound-Assisted Extraction Method Development

An ultrasonic bath (USC1200TH, Prolabo, Sion, Switzerland) consisting of a 300 × 240 × 200 mm (inside dimension) tank with an electric power of 400 W equal to an acoustic power of 1 W/cm^2^ and a maximum heating power of 400 W was used. A frequency controller selected the US frequency of the device, also equipped with a temperature regulator and an automatic digital timer. Each sample was suspended in 10 mL of extraction solvent and deposited in 50 mL quartz tubes with a vapor condenser. For extraction optimization, a Box–Behnken design was used, and the resulting response surface plots drawn with XLSTAT2019 software (Addinsoft, Paris, France). For this purpose, five variables (i.e., ultrasound frequency, extraction duration, aqueous ethanol (EtOH) concentration, extraction temperature, and liquid/solid ratio) were studied at three levels as shown in Table 1. The DOE (design of experiment) tool of XLSTAT 2019 (Addinsoft, Paris, France) was used to create and order the distinct observations (Table 2). Each observation was done in at least triplicate. The XLSTAT 2019 DOE analysis tool was used to determine the equation for the extraction and the 3D option to generate the necessary response surface plots (Addinsoft, Paris, France).

The optimal extraction conditions were: USAE with aqEtOH 63.81% (*v/v*) as a solvent for 39.19 min, at a US frequency of 29.96 kHz, at a temperature of 45 °C, and a solid to liquid ratio of 5.5 mg/mL (Figure S3).

### 4.4. Determination of the Total Flavonoid Content

Following extraction, each extract was centrifuged for 15 min at 5000× *g* (Heraeus Biofuge Stratos, Thermo Scientific, Illkirch, France), and the supernatant was filtered using a syringe filter (0.45 m, Merck Millipore, Molsheim, France) before analysis. The colorimetric aluminum trichloride (AlCl_3_) method was used to determine TFC [33]. A 200 µL mixture was made in a microplate using 20 µL of extract, 10 µL of potassium acetate 1 M, 10 µL of AlCl_3_ (10% (*w*/*v*)), and 160 µL of deionized water. A microplate reader (Multiskan GO, Thermo Fischer Scientific, Illkirch, France) was used to measure the absorbance at 415 nm after 30 min of incubation at 25 °C in the dark. TFC was expressed in mg/g dry weight (DW) of quercetin equivalent using a five-point calibration line (linearity range from 0 to 40 g/mL quercetin concentration with an R^2^ of 0.998).

### 4.5. HPLC-UV-DAD Analysis

Following extraction, each extract was centrifuged for 15 min at 5000× *g* (Heraeus Biofuge Stratos, Thermo Scientific, Illkirch, France), and the supernatant was filtered using a syringe filter (0.45 m, Merck Millipore, Molsheim, France) before analysis. HPLC was used to separate and identify the main isoflavonoids using a Varian system (Varian, Les Ulis, France) that included a Prostar 230 pump, Metachem Degasit, Prostar 410 autosampler, and Prostar 335 Photodiode Array Detector (PAD) and was controlled by Galaxie version 1.9.3.2 software (Varian, Les Ulis, France).

The separation was carried out on a Purospher RP-18 column (250 × 4.0 mm internal diameter; 5 µm) (Merck Chemicals, Molsheim, France) at a temperature of 40 °C. The validated separation conditions were described previously [39]. The mobile phase was a mixture of water and phosphoric acid (1000:1, *v/v*) (solvent A), and water, acetonitrile, and phosphoric acid (200:800:1, *v/v/v*) (solvent B). During the separation run (including 10 re-equilibration), the mobile phase composition varied according to a linear gradient as follows: B 0% (0 min) to 20% (5 min) to 100% (50 min) followed by 0% (60 min). Between each injection, a 10-min re-equilibration time was applied. The detection of compounds was set at 260 nm (corresponding to the λmax of the main compounds). Quantification was done based on assessment of the retention times of the commercial standard of daidzein (linear range 5–100 µg/mL, R^2^ = 0.999) and genistein (linear range 5–100 µg/mL, R^2^ = 0.999) (Merck, Saint-Quentin Fallavier, France). Because no commercial standard is available for cajanin and cajanol, their contents were semi-quantified using daidzein standard.

### 4.6. In Vitro Antioxidant Activities

The in vitro cell-free DPPH (2,2-diphenyl-1-picrylhydrazyl), FRAP (Ferric Reducing Antioxidant Power), ABTS (2,2-azinobis(3-ethylbenzthiazoline-6-sulphonic acid), and CUPRAC (Cupric Reducing Antioxidant Capacity) assays were used to evaluate the in vitro free radical scavenging activity of the samples using microplate-adapted protocols (Multiskan GO, Thermo Fischer Scientific, Illkirch, France) as described by Drouet et al. [40] and Tungmunnithum et al. [33].

### 4.7. Yeast Culture Conditions

The yeast strain DBY746 (MAT leu2-3,112 his31 trp1-289a ura3-52 GAI+) culture was started with frozen stock plated onto a YPD medium (yeast extract peptone dextrose) (Sigma-Aldrich, Saint-Quentin Fallavier, France). Extract (CAJ-USE at a final concentration of 1 mg/mL) and resveratrol (RES, positive control, at a final concentration of 10 µM) were dissolved in cell culture-grade dimethyl sulfoxide (DMSO; Sigma-Aldrich, Saint-Quentin Fallavier, France) and applied at a final DMSO concentration of 0.1% (*v/v*). Control yeast was inoculated with the same DMSO concentration. Survival was determined as previously described [65].

### 4.8. Cellular Antioxidant Assay

Yeast cells were first treated under the same conditions as mentioned above. Yeast cells were irradiated with a UV dose of 106.5 J/m2 UV-C (254 nm) under a Vilber VL-6.C filtered lamp (Thermo Fisher Scientific, Villebon-sur-Yvette, France), and incubated at 28 °C with orbital shaking at 120 rpm in the dark in complete 2.0% (*w*/*v*) glucose YPD medium (Sigma Aldrich, Saint-Quentin Fallavier, France) as previously described [65]. The same conditions were used to grow non-irradiated cells. Hour 0 of the oxidative stress experiment was considered irradiation.

The dihydrorhodamine-123 (DHR-123) fluorescent dye (Sigma-Aldrich, Saint-Quentin Fallavier, France) was used to assess the quantity of reactive oxygen and nitrogen species. Approximately 10^8^ yeast cells were washed twice in PBS, resuspended in PBS containing 0.4 M DHR-123, and incubated for 10 min in the dark at 28 °C in the presence of extract, RES, or DMSO (control cells). The fluorescence signal (ex = 505 nm, em = 535 nm) was measured using the VersaFluor Fluorimeter after two washes with PBS (Biorad, Marnes-la-Coquette, France).

### 4.9. Mitochondria Membrane Potential Evaluation

The mitochondria membrane potential (ΔΨm) was measured by monitoring the fluorescence of the specific probe 3,3′-dihexyloxacarbocyanine iodide (DiOC6(3); Sigma-Aldrich, Saint-Quentin Fallavier, France) as described by Tungmunnithum et al. [65]. At least six independent measurements were performed for each condition and the results were expressed as relative fluorescent units.

### 4.10. Gene Expression by RT-qPCR Analysis

Total RNA was extracted from the yeast cells at their exponential phase using the RiboPure RNA extraction kit (Thermo Scientific, Illkirch, France). Reverse transcription was performed using a SuperScript IV cDNA synthesis kit (Thermo Scientific, Illkirch, France) with oligo (dT) adaptor primer (Thermo Scientific, Illkirch, France), 1 unit of RiboLock (Thermo Scientific, Illkirch, France), and 5 mg of yeast total RNA quantified by the Quant-iT HR RNA assay and using a Qubit fluorimeter (Thermo Scientific, Illkirch, France).

Real-time PCR was performed with a PikoReal™ Real-Time PCR System (Thermo Scientific, Illkirch, France) using DyNAmo ColorFlash SYBR Green qPCR (Thermo Scientific, Illkirch, France) and specific primers. Primers used were: *SOD1*, forward: 5′-CACCATTTTCGTCCGTCTTT-3′, and reverse: 5′-TGGTTGTGTCTCTGCTGGTC-3′; *SOD2*, forward: 5′-CTCCGGTCAAATCAACGAAT-3′, and reverse: 5′-CCTTGGCCAGAAGATCTGAG-3′; *SIR2*, forward: 5′-CGTTCCCCAAGTCCTGATTA-3′, and reverse: 5′-CCACATTTTTGGGCTACCAT-3′; *TUB1*, forward: 5′-CCAAGGGCTATTTACGTGGA-3′, and reverse: 5′-GGTGTAATGGCCTCTTGCAT-3′. The qPCR parameters were as follows: an initial denaturation at 95 °C for 5 min, then 40 3-step cycles of 94 °C for 15 s, primer annealing at 55.4 °C for 10 s, and extension at 72 °C for 20 s. After 40 cycles, a final extension phase was carried out for 90 s at 72 °C. Observation of a single peak in the melting curve obtained after amplification indicated the existence of a single amplicon. The amounts of mRNA *SIR2*, *SOD1*, and *SOD2* were normalized to that of *TUB1*.

Expression levels were calculated and normalized using the 2^−ΔΔCt^ method. Reactions were made in four biological replicates, and two technical replicates were performed for each measurement.

### 4.11. Enzymatic SIRT1/SIR2 and Total SOD Activity Determinations

For protein extraction, approximately 10^8^ yeast cells were washed three times with PBS. Then, 1 mL of PBS was added, and the mixture was subjected to three freeze and thaw cycles using liquid nitrogen. The cell lysate was then centrifuged at 10,000× *g* at 4 °C for 15 min, and the supernatant was used to prepare the sample solution by dilution with PBS. Proteins were quantified using the Qubit Protein Assay Kit following the manufacturer’s instructions and using the Qubit fluorimeter (Thermo Scientific, Illkirch, France).

Total SOD activity was measured using the Superoxide Dismutase Activity kit following the manufacturer’s instructions (Thermo Scientific, Illkirch, France).

SIRT1/SIR2 activity was determined using the SIRT1 Assay Kit (Sigma-Aldrich, Saint-Quentin Fallavier, France) following the manufacturer’s instructions and using a Versafluor fluorimeter (Biorad, Marnes-la-Coquette, France).

### 4.12. Cellular Oxidative Stress Products

The membrane lipid peroxide was measured using thiobarbituric acid (TBA; Sigma Aldrich, Saint-Quentin Fallavier, France). Approximately 10^8^ yeast cells were crushed in double distilled water and centrifuged at 10,000× *g* for 10 min. The supernatant (75 µL) was combined with 25 µL of SDS (3% *w/v*), 50 L of TBA (3% *w/v*) produced in 50 mM NaOH, and 50 µL of HCl (23% *v/v*). Between each addition, the mixture was thoroughly mixed. The resulting combination was heated to 80 °C for 20 min, then cooled on ice before measuring the absorbance at 532 nm (A532). Absorbance at 600 nm (i.e., non-specific absorbance measurement) was removed. A standard curve was prepared using 1,1,3,3, tetramethoxypropane to measure the concentrations of TBARS in the samples.

Total proteins were extracted from about 10^8^ yeast cells as described above (Section 4.11). The protein carbonyl content was determined using a Protein Carbonyl ELISA kit following the manufacturer’s instructions (Cell BioLabs, Paris, France).

DNA was extracted from about 10^8^ yeast cells with a Yeast DNA Extraction Reagent Kit following the manufacturer’s instructions (Thermo Scientific, Illkirch, France) and the 8-oxo-guanine content was determined with the 8-OHdG DNA Damage ELISA kit following the manufacturer’s instructions (Cell BioLabs, Paris, France).

### 4.13. Statistical Analysis

Statistical analyses were performed with XLSTAT 2019 suite (Addinsoft, Paris, France). Data composed of at least three independent replicates were presented using the means and standard deviations. A Student *t*-test was carried out for statistical comparative analysis. Significant thresholds at *p* < 0.05, 0.01, and 0.001 were represented by *, **, and ***, respectively. Different letters were used to indicate significant thresholds at *p* < 0.05.

## 5. Conclusions

The current work is the first and fundamental study that clearly showed that ultrasound-assisted extraction methodology is a potential green extraction method that can provide ca 70% higher (iso)flavonoids extraction compared with the conventional extraction with the same extraction duration, whereas its biological antioxidant activities were preserved. It is clearly evidenced that pigeon pea seeds are a potential raw material for cosmetic and other phytopharmaceutical applications. The cellular antioxidant and anti-aging potential of pigeon pea seed extract obtained under optimal USAE were high, resulting in lower levels of malondialdehyde but also lower levels of protein carbonylation and 8-oxo-guanine formation, thus demonstrating that the extract is an effective protector against UV-induced oxidative stress. This extract also acts as an effective activator of the cell longevity protein (SIR2/SIRT1) at the cellular level using yeast as a model. This study corroborates the use of pigeon pea seeds and USAE technology to produce antioxidant and anti-aging (iso)flavonoids-rich sources for the cosmetic and phytopharmaceutical industries. We believe that future (cyto)toxicity testing in humans will not be a problem due to the edible nature of the starting raw material, thus allowing this fast, efficient, and reproducible extraction procedure to be used for the development of new innovative beauty products with pigeon pea seed extract.

## Figures and Tables

**Table 1:** The five independent variables identities, code units, coded levels, and actual experimental values.

Independent Variable	Code Unit	Coded Variable Levels		
		−1	0	+1
Extraction duration (min)	X_1_	20	40	60
US frequency (kHz)	X_2_	0	22.5	45
Ethanol concentration (% *v/v*) ^1^	X_3_	50	75	100
Extraction temperature (°C)	X_4_	30	45	60
Liquid/solid ratio (mg/mL) ^2^	X_5_	1	5.5	10

^1^ in % of the ethanol concentration in the mixture with ultrapure water (HPLC grade); ^2^ in mg of pigeon pea seed dry material per mL of solvent.

**Table 2:** Results of experimental and predicted TFC.

Run ID	Run Order	X_1_	X_2_	X_3_	X_4_	X_5_	Experimental TFC (mg/100 g DM)	Predicted TFC (mg/100 g DM)
Obs1	21	−1	−1	0	0	0	23.94 ± 1.35	23.28
Obs2	15	+1	−1	0	0	0	26.05 ± 2.68	23.23
Obs3	1	−1	+1	0	0	0	30.46 ± 1.71	32.47
Obs4	16	+1	+1	0	0	0	30.66 ± 0.37	30.51
Obs5	33	−1	0	−1	0	0	25.60 ± 1.62	23.81
Obs6	28	+1	0	−1	0	0	24.76 ± 2.31	22.28
Obs7	27	−1	0	+1	0	0	29.56 ± 0.03	29.41
Obs8	17	+1	0	+1	0	0	29.76 ± 0.67	28.93
Obs9	34	−1	0	0	−1	0	34.24 ± 0.12	33.65
Obs10	9	+1	0	0	−1	0	32.27 ± 0.94	33.64
Obs11	20	−1	0	0	+1	0	36.04 ± 0.50	35.76
Obs12	13	+1	0	0	+1	0	32.08 ± 1.32	33.76
Obs13	4	−1	0	0	0	−1	33.33 ± 0.25	33.95
Obs14	24	+1	0	0	0	−1	31.44 ± 1.46	32.94
Obs15	3	−1	0	0	0	+1	31.70 ± 0.36	32.54
Obs16	8	+1	0	0	0	+1	29.81 ± 1.57	31.54
Obs17	29	0	−1	−1	0	0	24.39 ± 2.42	22.33
Obs18	10	0	+1	−1	0	0	27.04 ± 1.10	25.92
Obs19	11	0	−1	+1	0	0	25.69 ± 2.24	23.80
Obs20	37	0	+1	+1	0	0	37.64 ± 0.92	36.69
Obs21	30	0	−1	0	−1	0	29.46 ± 3.63	33.37
Obs22	2	0	+1	0	−1	0	37.07 ± 0.85	36.08
Obs23	36	0	−1	0	+1	0	26.59 ± 1.87	28.96
Obs24	19	0	+1	0	+1	0	45.26 ± 2.61	42.72
Obs25	22	0	−1	0	0	−1	31.20 ± 2.70	32.53
Obs26	35	0	+1	0	0	−1	33.90 ± 2.81	36.52
Obs27	40	0	−1	0	0	+1	27.07 ± 0.48	26.87
Obs28	26	0	+1	0	0	+1	38.26 ± 0.60	39.36
Obs29	25	0	0	−1	−1	0	30.11 ± 2.53	32.59
Obs30	12	0	0	+1	−1	0	31.94 ± 2.44	34.33
Obs31	7	0	0	−1	+1	0	27.75 ± 1.70	29.32
Obs32	41	0	0	+1	+1	0	38.24 ± 1.61	39.83
Obs33	5	0	0	−1	0	−1	27.70 ± 1.68	29.66
Obs34	31	0	0	+1	0	−1	36.62 ± 0.05	36.86
Obs35	39	0	0	−1	0	+1	27.90 ± 1.53	29.33
Obs36	18	0	0	+1	0	+1	34.67 ± 0.20	34.37
Obs37	23	0	0	0	−1	−1	45.01 ± 5.53	39.80
Obs38	6	0	0	0	+1	−1	45.98 ± 3.32	42.91
Obs39	38	0	0	0	−1	+1	43.75 ± 3.28	40.39
Obs40	32	0	0	0	+1	+1	40.73 ± 1.07	39.51
Obs41 *	14	0	0	0	0	0	41.80 ± 1.15 *	41.80

Values are the mean ± RSD of 3 independent replicates, except for the central point (*), which is the mean ± RSD of 10 independent replicates.

**Table 3:** ANOVA of the predicted model.

Source	Sum of Square	df	Mean of Square	*F*-Value	*p*-Value
Model	2003.80	20	100.19	19.516	<0.0001
Lack of fit	154.01	30	5.13	-	-
Residual	154.01	30	5.13	-	-
Pure Error	0.000	0	-	-	-
Cor. Error	2157.81	50	-	-	-
R^2^	0.929				
R^2^ adj	0.881				
CV %	0.715				

df: degree of freedom; Cor. Error: corrected error; R^2^: determination coefficient; R^2^ adj: adjusted R^2^; CV variation coefficient value.

**Table 4:** Statistical analysis of the regression coefficients.

Source	Value	SD	*t*	*p* > |*t*|
Constant	41.797	0.683	61.181	<0.0001 ***
X_1_	−0.502	0.566	−0.886	0.383
X_2_	4.119	0.566	7.272	<0.0001 ***
X_3_	3.062	0.566	5.405	<0.0001 ***
X_4_	0.558	0.566	0.984	0.333
X_5_	−0.705	0.566	−1.245	0.223
X_1_^2^	−7.751	0.701	−11.062	<0.0001 ***
X_2_^2^	−6.671	0.701	−9.521	<0.0001 ***
X_3_^2^	−7.938	0.701	−11.329	<0.0001 ***
X_4_^2^	0.159	0.701	0.227	0.822
X_5_^2^	−1.303	0.701	−1.860	0.073
X_1_X_2_	−0.478	1.133	−0.422	0.676
X_1_X_3_	0.260	1.133	0.229	0.820
X_1_X_4_	−0.498	1.133	−0.440	0.663
X_1_X_5_	0.001	1.133	0.001	0.999
X_2_X_3_	2.325	1.133	2.052	0.049 *
X_2_X_4_	2.763	1.133	2.439	0.021 *
X_2_X_5_	2.124	1.133	1.875	0.071
X_3_X_4_	2.190	1.133	1.933	0.063
X_3_X_5_	−0.539	1.133	−0.475	0.638
X_4_X_5_	−0.998	1.133	−0.881	0.385

SD standard error; *** significant *p* < 0.001; * significant *p* < 0.05.

**Table 5:** Comparison of the optimized USAE procedure with the conventional heat reflux method (HRE, i.e., without US) in terms of the flavonoids extraction capacity and antioxidant activity.

	TFC (mg/100 g DM)	Daidzein (mg/100 g DM)	Genistein (mg/100 g DM)	Cajanin (mg/100 g DM)	Cajanol (mg/100 g DM)	DPPH (µmol TEAC/g DM)
Optimized USAE	48.96 ± 0.54 ^a^	9.03 ± 0.14 ^a^	0.78 ± 0.02 ^a^	18.11 ± 0.27 ^a^	11.64 ± 0.17 ^a^	514.68 ± 7.88 ^a^
Conventional HRE	28.77 ± 4.01 ^b^	5.12 ± 0.41 ^b^	0.32 ± 0.07 ^b^	11.24 ± 0.33 ^b^	6.31 ± 0.48 ^b^	172.52 ± 11.47 ^b^

Values are means ± standard deviations (SD) of 6 independent experiments. Different letters represent significant differences (*p* < 0.05).

## Data Availability

All the data supporting the findings of this study are included in this article.

## Supplementary Materials

The following are available online. Figure S1. Basic structure of A. flavonoid and B. isoflavonoid, Figure S2: Yeast cells’ survival rate under UV stress, Figure S3. Workflow of the optimized USAE of (iso)flavonoids from pigeon pea seeds, Table S1: TFC and in vitro cell-free antioxidant activity values in the 41 BBD observations.

## Sample Availability

Samples presented in this study are available from the authors upon request.

## References

[1] Tungmunnithum D., Hano C. (2020). Cosmetic potential of *Cajanus cajan* (L.) millsp: Botanical data, traditional uses, phytochemistry and biological activities. Cosmetics.

[2] Ahsan R., Islam M. (2009). In vitro antibacterial screening and toxicological study of some useful plants (*Cajanus cajan*). Eur. J. Sci. Res..

[3] Ambasta S.P. (2004). The Useful Plants of India.

[4] Kiwia A., Kimani D., Harawa R., Jama B., Sileshi G.W. (2019). Sustainable Intensification with Cereal-Legume Intercropping in Eastern and Southern Africa. Sustainability.

[5] Owens J.D., Astuti M.K., Owens J.D. (2015). Tempe and related products. Indigenous Fermented Foods of Southeast Asia.

[6] Fuller D.Q., Murphy C., Kingwell-Banham E., Castillo C.C., Naik S. (2019). *Cajanus cajan* (L.) Millsp. origins and domestication: The South and Southeast Asian archaeobotanical evidence. Genet. Resour. Crop. Evol..

[7] Upadhyay B., Parveen, Dhaker A.K., Kumar A. (2010). Ethnomedicinal and ethnopharmaco—Statistical studies of Eastern Rajasthan, India. J. Ethnopharmacol.

[8] Darbyshire I., Kordofani M., Farag I., Candiga R., Pickering H. (2015). The Plants of Sudan and South. Sudan.

[9] Plants of the World. http://www.plantsoftheworldonline.org/taxon/urn:lsid:ipni.org:names:1152177-2.

[10] Wu Z., Raven P.H. (2010). Cajanus. Flora of China.

[11] Ganesan S. (2008). Traditional oral care medicinal plants survey of Tamil nadu. Nat. Prod. Rad..

[12] Al-Saeedi A.H., Amzad Hossain M. (2015). Total phenols, total flavonoids contents and free radical scavenging activity of seeds crude extracts of pigeonpea traditionally used in Oman for the treatment of several chronic diseases. Asian Pac. J. Trop. Dis..

[13] Hassan E.M., Matloub A., Aboutabl M.E., Ibrahim N.A., Mohamed S. (2015). Assessment of anti-inflammatory, antinociceptive, immunomodulatory, and antioxidant activities of *Cajanus cajan* L. seeds cultivated in Egypt and its phytochemical composition. Pharm. Biol..

[14] Uchegbu N.N., Ishiwu C.N. (2016). Germinated Pigeon Pea (Cajanus cajan): A novel diet for lowering oxidative stress and hyperglycemia. Food Sci. Nutr..

[15] Sarkar R., Mandal N. (2012). Hydroalcoholic extracts of Indian medicinal plants can help in amelioration from oxidative stress through antioxidant properties. J. Complement. Integr. Med..

[16] Wei Z.-F., Jin S., Luo M., Pan Y.-Z., Li T.-T., Qi X.-L., Efferth T., Fu Y.-J., Zu Y.-G. (2013). Variation in Contentsof Main Active Components and Antioxidant Activity in Leaves of Dierent Pigeon Pea Cultivars during Growth. J. Agric. Food Chem..

[17] Mahitha B., Archana P., Ebrahimzadeh M.H., Srikanth K., Rajinikanth M., Ramaswamy N. (2015). In vitro Antioxidant and Pharmacognostic Studies of Leaf Extracts of *Cajanus cajan* (L.) Millsp. Indian J. Pharm. Sci..

[18] Hano C., Tungmunnithum D. (2020). Plant Polyphenols, More than Just Simple Natural Antioxidants: Oxidative Stress, Aging and Age-Related Diseases. Medicines.

[19] Liggins J., Bluck L.J.C., Runswick S., Atkinson C., Coward W.A., Bingham S.A. (2000). Daidzein and genistein contents of vegetables. Br. J. Nutr..

[20] Vo T.-L.T., Yang N.-C., Yang S.-E., Chen C.-L., Wu C.-H., Song T.-Y. (2020). Effects of *Cajanus cajan* (L.) millsp. roots extracts on the antioxidant and anti-inflammatory activities. Chin. J. Physiol..

[21] Zheng X., Wang X., Lan Y., Shi J., Jun S., Liu C. (2009). Application of response surface methodology to optimize microwave-assisted extraction of silymarin from milk thistle seeds. Sep. Purif. Technol.

[22] Fliniaux O., Corbin C., Ramsay A., Renouard S., Beejmohun V., Doussot J., Falguières A., Ferroud C., Lamblin F., Lainé E. (2014). Microwave-Assisted Extraction of Herbacetin Diglucoside from Flax (*Linum usitatissimum* L.) Seed Cakes and Its Quantification using an RP-HPLC-UV System. Molecules.

[23] Benthin B., Danz H., Hamburger M. (1999). Pressurized liquid extraction of medicinal plants. J. Chromatogr. A.

[24] Renouard S., Hano C., Corbin C., Fliniaux O., Lopez T., Montguillon J., Barakzoy E., Mesnard F., Lamblin F., Lainé E. (2010). Cellulase-assisted release of secoisolariciresinol from extracts of flax (*Linum usitatissimum*) hulls and whole seeds. Food Chem..

[25] Ozkur M.K., Bozkurt M.S., Balabanli B., Aricioglu A., Ilter N., Gurer M.A., Inaloz H.S. (2002). The effect of EGb 761 on lipid peroxide levels and superoxide dismutase activity in sunburn. Photodermatol. Photoimmunol. Photomed..

[26] Olas B. (2017). The multifunctionality of berries toward blood platelets and the role of berry phenolics in cardiovascular disorders. Platelets.

[27] Oki T., Masuda M., Furuta S., Nishiba Y., Terahara N., Suda A.I. (2002). Involvement of Anthocyanins and Other Phenolic Compounds in Radical Scavenging Activity of Purple-Fleshed Sweet Potato Cultivars. Food Chem. Toxicol..

[28] Okpuzor J., Ogbunugafor H., Kareem G.K., Igwo-Ezikpe M.N. (2009). In vitro investigation of antioxidant phenolic compounds in extracts of *Senna alata*. Res. J. Phytochem..

[29] Nagja T., Vimal K., Sanjeev A. (2016). Myristica Fragrans: A comprehensive review. Int. J. Pharm. Pharm. Sci..

[30] Drouet S., Leclerc E.A., Garros L., Tungmunnithum D., Kabra A., Abbasi B.H., Lain É., Hano C. (2019). A Green Ultrasound-Assisted Extraction Optimization of the Natural Antioxidant and Anti-Aging Flavonolignans from Milk Thistle *Silybum marianum* (L.) Gaertn. Fruits for Cosmetic Applications. Antioxidants.

[31] Giordano M., Pinela J., Dias M.I., Calhelha R.C., Stojković D., Soković M., Tavares D., Cánepa A.L., Ferreira I.C.F.R., Caleja C. (2021). Ultrasound-Assisted Extraction of Flavonoids from Kiwi Peel: Process Optimization and Bioactivity Assessment. Appl. Sci..

[32] Lee M., Lin C. (2007). Comparison of techniques for extraction of isoflavones from the root of Radix Puerariae: Ultrasonic and pressurized solvent extractions. Food Chem..

[33] Tungmunnithum D., Drouet S., Kabra A., Hano C. (2020). Enrichment in Antioxidant Flavonoids of Stamen Extracts from *Nymphaea lotus* L. Using Ultrasonic-Assisted Extraction and Macroporous Resin Adsorption. Antioxidants.

[34] Tiwari B.K. (2015). Ultrasound: A clean, green extraction technology. TrAC Trends Anal. Chem..

[35] Chemat F., Abert-Vian M., Fabiano-Tixier A.S., Strube J., Uhlenbrock L., Gunjevic V., Cravotto G. (2019). Green extraction of natural products. Origins, current status, and future challenges. Trends Anal. Chem..

[36] Lavilla I., Bendicho C. (2017). Fundamentals of Ultrasound-Assisted Extraction. Water Extraction of Bioactive Compounds: From Plants to Drug Development.

[37] Nazir M., Tungmunnithum D., Bose S., Drouet S., Garros L., Giglioli-Guivarc’h N., Abbasi B.H., Hano C. (2019). Differential Production of Phenylpropanoid Metabolites in Callus Cultures of *Ocimum basilicum* L. With Distinct in Vitro Antioxidant Activities and in Vivo Protective Effects against UV stress. J. Agric. Food Chem..

[38] Prior R.L., Wu X., Schaich K. (2005). Standardized Methods for the Determination of Antioxidant Capacity and Phenolics in Foods and Dietary Supplements. J. Agric. Food Chem..

[39] Nakamura Y., Kaihara A., Yoshii K., Tsumura Y., Ishimitsu S., Tonogai Y. (2001). Content and composition of isoflavonoids in mature or immature beans and bean sprouts consumed in Japan. J. Health Sci..

[40] Drouet S., Doussot J., Garros L., Mathiron D., Bassard S., Favre-Réguillon A., Molinié R., Lainé É., Hano C. (2018). Selective Synthesis of 3-O-Palmitoyl-Silybin, a New-to-Nature Flavonolignan with Increased Protective Action against Oxidative Damages in Lipophilic Media. Molecules.

[41] Corbin C., Fidel T., Leclerc E.A., Barakzoy E., Sagot N., Falguiéres A., Renouard S., Blondeau J., Ferroud C., Doussot J. (2015). Development and validation of an efficient ultrasound assisted extraction of phenolic compounds from flax (*Linum usitatissimum* L.) seeds. Ultrason. Sonochem..

[42] Tungmunnithum D., Garros L., Drouet S., Renouard S., Lainé E., Hano C. (2019). Green Ultrasound Assisted Extraction of trans Rosmarinic Acid from *Plectranthus scutellarioides* (L.) R.Br. Leaves. Plants.

[43] Hostettmann K., Hostettmann M. (1982). Isolation Techniques for Flavonoids. The Flavonoids.

[44] Ranjha M.M.A.N., Irfan S., Lorenzo J.M., Shafique B., Kanwal R., Pateiro M., Arshad R.N., Wang L., Nayik G.A., Roobab U. (2021). Sonication, a Potential Technique for Extraction of Phytoconstituents: A Systematic Review. Processes.

[45] Lopez T., Corbin C., Falguieres A., Doussot J., Montguillon J., Hagège D., Hano C., Lainé É. (2016). Secondary metabolite accumulation, antibacterial and antioxidant properties of in vitro propagated *Clidemia hirta* L. extracts are influenced by the basal culture medium. Comptes Rendus Chim..

[46] Bourgeois C., Leclerc É.A., Corbin C., Doussot J., Serrano V., Vanier J.-R., Seigneuret J.-M., Auguin D., Pichon C., Lainé É. (2016). Nettle (*Urtica dioica* L.) as a source of antioxidant and anti-aging phytochemicals for cosmetic applications, L’ortie (*Urtica dioica* L.), une source de produits antioxidants et phytochimiques anti-âge pour des applications en cosmétique. Comptes Rendus Chim..

[47] Tungmunnithum D., Elamrani A., Abid M., Drouet S., Kiani R., Garros L., Kabra A., Addi M., Hano C. (2020). A Quick, Green and Simple Ultrasound-Assisted Extraction for the Valorization of Antioxidant Phenolic Acids from Moroccan Almond Cold-Pressed Oil Residues. Appl. Sci..

[48] Renouard S., Corbin C., Colas C., Fidel T., Lopez T., Leclerc E.A., Hendrawati O., Falguières A., Doussot J., Ferroud C. (2015). Aerial parts of Callitris species as a rich source of deoxypodophyllotoxin. Ind. Crops Prod..

[49] Terpinc P., Polak T., Makuc D., Ulrih N.P., Abramovič H. (2012). The occurrence and characterisation of phenolic compounds in Camelina sativa seed, cake and oil. Food Chem..

[50] Ferreira S.L.C., Bruns R.E., Ferreira H.S., Matos G.D., David J.M., Brandão G.C., da Silva E.G.P., Portugal L.A., dos Reis P.S., Souza A.S. (2007). Box-Behnken design: An alternative for the optimization of analytical methods. Anal. Chim. Acta.

[51] Ferreira S.L.C., Silva Junior M.M., Felix C.S.A., da Silva D.L.F., Santos A.S., Santos Neto J.H., de Souza C.T., Cruz Junior R.A., Souza A.S. (2019). Multivariate optimization techniques in food analysis—A review. Food Chem..

[52] Anastas P.T., Warner J.C. (1998). Green Chemistry: Theory and Practice.

[53] Ameer K., Shahbaz H.M., Kwon J.H. (2017). Green Extraction Methods for Polyphenols from Plant Matrices and Their Byproducts: A Review. Compr. Rev. Food Sci. Food Saf..

[54] Garros L., Drouet S., Corbin C., Decourtil C., Fidel T., de Lacour J.L., Leclerc E.A., Renouard S., Tungmunnithum D., Doussot J. (2018). Insight into the influence of cultivar type, cultivation year, and site on the lignans and related phenolic profiles, and the health-promoting antioxidant potential of flax (*Linum usitatissimum* L.) seeds. Molecules.

[55] Drouet S., Abbasi B.H., Falguières A., Ahmad W., Sumaira, Ferroud C., Doussot J., Vanier J.R., Lainé E., Hano C. (2018). Single Laboratory Validation of a Quantitative Core Shell-Based LC Separation for the Evaluation of Silymarin Variability and Associated Antioxidant Activity of Pakistani Ecotypes of Milk Thistle (*Silybum Marianum* L.). Molecules.

[56] Hano C., Corbin C., Drouet S., Quéro A., Rombaut N., Savoire R., Molinié R., Thomasset B., Mesnard F., Lainé E. (2017). The lignan (+)-secoisolariciresinol extracted from flax hulls is an effective protectant of linseed oil and its emulsion against oxidative damage. Eur. J. Lipid Sci. Technol..

[57] Steels E.L., Learmonth R.P., Watson K. (1994). Stress tolerance and membrane lipid unsaturation in Saccharomyces cerevisiae grown aerobically or anaerobically. Microbiology.

[58] Wolak N., Kowalska E., Kozik A., Rapala-Kozik M. (2014). Thiamine increases the resistance of baker’s yeast *Saccharomyces cerevisiae* against oxidative, osmotic and thermal stress, through mechanisms partly independent of thiamine diphosphate-bound enzymes. FEMS Yeast Res..

[59] Rice-Evans C.A., Miller N.J., Paganga G., Catherine A.R.-E., Nicholas J.M., George P. (1996). Structure-antioxidant activity relationships of flavonoids and phenolic acids. Free Radic. Biol. Med..

[60] Lacza Z., Pankotai E., Csordás A., Gero D., Kiss L., Horváth E.M., Kollai M., Busija D.W., Szabó C. (2006). Mitochondrial NO and reactive nitrogen species production: Does mtNOS exist?. Nitric Oxide.

[61] Merksamer P.I., Liu Y., He W., Hirschey M.D., Chen D., Verdin E. (2013). The sirtuins, oxidative stress and aging: An emerging link. Aging.

[62] Semchyshyn H.M., Lozinska L.M. (2012). Fructose protects baker’s yeast against peroxide stress: Potential role of catalase and superoxide dismutase. FEMS Yeast Res..

[63] Tanno M., Kuno A., Yano T., Miura T., Hisahara S., Ishikawa S., Shimamoto K., Horio Y. (2010). Induction of manganese superoxide dismutase by nuclear translocation and activation of SIRT1 promotes cell survival in chronic heart failure. J. Biol. Chem..

[64] Daitoku H., Hatta M., Matsuzaki H., Aratani S., Ohshima T., Miyagishi M., Nakajima T., Fukamizu A. (2004). Silent information regulator 2 potentiates Foxo1-mediated transcription through its deacetylase activity. Proc. Natl. Acad. Sci. USA.

[65] Tungmunnithum D., Abid M., Elamrani A., Drouet S., Addi M., Hano C. (2020). Almond Skin Extracts and Chlorogenic Acid Delay Chronological Aging and Enhanced Oxidative Stress Response in Yeast. Life.

[66] Sun K., Cao S., Pei L., Matsuura A., Xiang L., Qi J. (2013). A steroidal saponin from *Ophiopogon japonicus* extends the lifespan of yeast via the pathway involved in SOD and UTH1. Int. J. Mol. Sci..

[67] Anna Malinowska M., Billet K., Drouet S., Munsch T., Unlubayir M., Tungmunnithum D., Giglioli-Guivarc’h N., Hano C., Lanoue A. (2020). Grape Cane Extracts as Multifunctional Rejuvenating Cosmetic Ingredient: Evaluation of Sirtuin Activity, Tyrosinase Inhibition and Bioavailability Potential. Molecules.

[68] Williams G.M., Iatropoulos M.J., Whysner J. (1999). Safety Assessment of Butylated Hydroxyanisole and Butylated Hydroxytoluene as Antioxidant Food Additives. Food Chem. Toxicol..

