# Supplementary material for: Green Extraction of Antioxidant Flavonoids from Pigeon Pea (Cajanus cajan (L.) Millsp.) Seeds and Its Antioxidant Potentials Using Ultrasound-Assisted Methodology
Source: Molecules. 2021 Dec 13;26(24):7557. doi: 10.3390/molecules26247557 (PMC8703396; doi:10.3390/molecules26247557)
Supplement: Supplementary file 1 [file molecules-26-07557-s001.zip › molecules-1481511-supplementary.pdf]

Supplementary Materials

Green Extraction of Antioxidant Flavonoids from Pigeon Pea (*Cajanus cajan* (L.) Millsp) Seeds and Its Antioxidant Potentials using Ultrasound-Assisted Methodology

Duangjai Tungmunnithum [1,2,3,*], Samantha Drouet [2], Jose Manuel Lorenzo [4,5] and Christophe Hano [2,3,*]

1 Department of Pharmaceutical Botany, Faculty of Pharmacy, Mahidol University, Bangkok 10400, Thailand; duangjai.tun@mahidol.ac.th (D.T.)
2 Laboratoire de Biologie des Ligneux et des Grandes Cultures, INRAE USC1328, Campus Eure et Loir, Orleans University, 28000 Chartres, France; duangjai.tun@mahidol.ac.th (D.T.) ; samantha.drouet@univ-orleans.fr (S.D.) ; hano@univ-orleans.fr (C.H.)
3 Le Studium Institue for Advanced Studies, 1 Rue Dupanloup, 45000 Orléans, France; duangjai.tun@mahidol.ac.th (D.T.) ; hano@univ-orleans.fr (C.H.)
4 Centro Tecnológico de la Carne de Galicia, Adva. Galicia n° 4, Parque Tecnológico de Galicia, San Cibrao das Viñas, 32900 Ourense, Spain; jmlorenzo@ceteca.net (J.M.L.)
6 Área de Tecnología de los Alimentos, Facultad de Ciencias de Ourense, Universidad de Vigo, 32004 Ourense, Spain
* Correspondence: duangjai.tun@mahidol.ac.th (D.T.); hano@univ-orleans.fr (C.H.)

Content:

Table S1. TFC and *in vitro* cell-free antioxidant activity values in the 41 BBD observations.

Figure S1. Basic structure of A. flavonoid and B. isoflavonoid.

Figure S2. Yeast cells survival rate under UV stress.

Figure S3. Workflow of the optimized USAE of (iso)flavonoids from pigeon pea seeds.

Table S1. TFC and *in vitro* cell-free antioxidant activity values in the 41 BBD observations.

	TFC (mg/g DM)		ABTS (μmol TEAC/mg DM)		DPPH (μmol TEAC/mg DM)		CUPRAC (μmol TEAC/mg DM)		FRAP (μmol TEAC/mg DM)	
	Mean	SD	Mean	SD	Mean	SD	Mean	SD	Mean	SD
**Obs1**	23.94	1.35	169.83	3.13	160.04	1.25	30.69	0.95	31.83	1.90
**Obs2**	26.05	2.68	161.94	2.16	156.42	0.72	53.98	3.65	55.64	1.44
**Obs3**	30.46	1.71	176.47	1.44	216.54	3.74	70.38	1.44	90.59	1.44
**Obs4**	30.66	0.37	178.13	3.30	247.74	1.25	70.38	6.50	91.42	1.90
**Obs5**	25.60	1.62	119.59	4.99	152.35	4.37	48.79	2.59	51.07	1.25
**Obs6**	24.76	2.31	108.37	1.21	150.09	1.44	38.17	3.81	43.18	0.72
**Obs7**	29.56	0.03	173.98	1.90	188.06	0.72	67.68	1.25	68.93	1.90
**Obs8**	29.76	0.67	174.81	4.00	190.32	0.72	67.89	0.72	79.76	2.16
**Obs9**	34.24	0.12	200.14	1.90	294.75	2.16	95.99	1.65	116.82	0.72
**Obs10**	32.27	0.94	194.74	1.38	287.52	1.25	82.95	3.07	104.33	1.90
**Obs11**	36.04	0.50	203.46	5.62	303.34	0.72	110.76	0.72	121.65	4.72
**Obs12**	32.08	1.32	187.68	7.18	284.36	2.88	84.95	1.30	103.50	1.25
**Obs13**	33.33	0.25	195.99	1.44	288.42	3.30	89.16	3.75	105.16	0.72
**Obs14**	31.44	1.46	180.62	1.25	258.59	3.81	75.67	2.52	72.25	1.25
**Obs15**	31.70	0.36	181.04	1.44	262.20	4.00	73.91	1.54	102.67	7.30
**Obs16**	29.81	1.57	174.81	7.08	207.50	1.25	68.10	3.89	69.76	2.49
**Obs17**	24.39	2.42	82.22	8.98	106.77	2.88	33.39	2.36	35.35	0.42
**Obs18**	27.04	1.10	165.68	4.49	156.87	2.88	59.89	4.24	63.95	1.44
**Obs19**	25.69	2.24	144.08	1.50	152.80	3.81	50.24	2.00	54.39	2.59
**Obs20**	37.64	0.92	230.04	0.72	329.20	0.72	133.88	6.66	138.55	7.30
**Obs21**	29.46	3.63	172.74	4.00	173.15	0.72	66.64	3.81	63.51	0.60
**Obs22**	37.07	0.85	224.22	1.88	316.45	0.72	121.18	2.00	144.31	0.72
**Obs23**	26.59	1.87	164.02	4.72	156.42	3.30	54.39	2.19	60.62	1.44
**Obs24**	45.26	2.61	282.43	3.35	429.92	2.97	218.62	1.04	275.37	1.45
**Obs25**	31.20	2.70	178.96	2.59	252.26	1.90	72.67	2.16	97.83	3.60
**Obs26**	33.90	2.81	197.65	5.62	288.88	0.72	92.57	10.28	115.57	1.44
**Obs27**	27.07	0.48	165.68	3.30	158.68	1.90	63.74	3.81	65.61	1.44
**Obs28**	38.26	0.60	230.87	0.72	340.87	1.25	149.56	4.21	159.45	3.74
**Obs29**	30.11	2.53	175.64	2.49	216.09	1.25	69.55	2.81	85.17	1.44
**Obs30**	31.94	2.44	182.29	3.60	270.34	1.44	74.33	3.65	93.08	1.90
**Obs31**	27.75	1.70	166.92	3.30	165.91	1.25	65.40	7.58	67.68	1.90
**Obs32**	38.34	1.61	231.70	1.25	346.74	75.58	169.76	4.72	168.17	5.03
**Obs33**	27.70	1.68	166.51	1.44	164.56	0.72	64.15	0.95	77.68	1.90
**Obs34**	36.62	0.05	204.29	1.25	304.70	0.72	118.13	1.30	138.48	6.59
**Obs35**	27.90	1.53	167.34	1.90	172.24	0.72	68.23	1.65	65.10	1.90
**Obs36**	34.67	0.20	200.97	2.59	295.21	9.73	96.61	9.67	117.23	1.25
**Obs37**	45.01	5.53	264.37	5.19	423.60	2.66	213.85	2.29	260.77	2.81
**Obs38**	45.98	3.32	292.32	3.60	448.46	3.72	223.34	2.83	303.95	5.53
**Obs39**	43.75	3.28	234.19	1.25	412.75	2.74	201.87	1.45	226.58	2.34
**Obs40**	40.73	1.07	233.36	1.44	346.92	1.68	159.10	3.62	201.74	1.89
**Obs41**	41.80	1.15	233.36	1.90	351.72	7.88	178.20	1.62	226.30	3.79

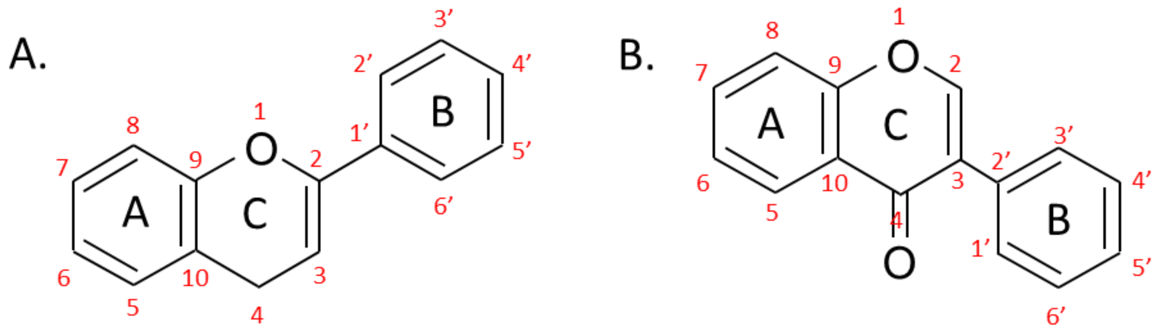

Figure S1. Basic structure of A. flavonoid and B. isoflavonoid.

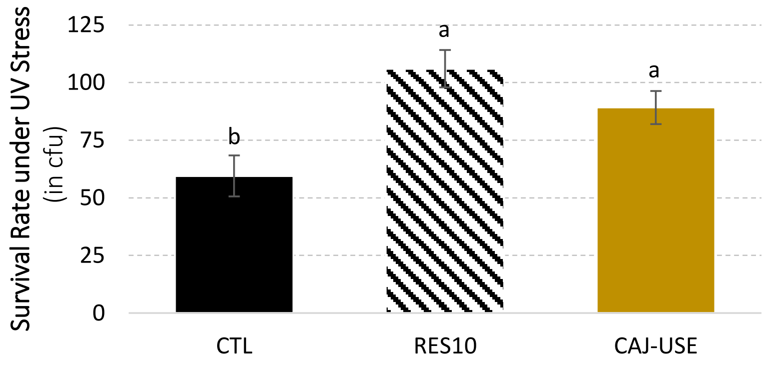

Figure S2. Yeast cells survival rate under UV stress.

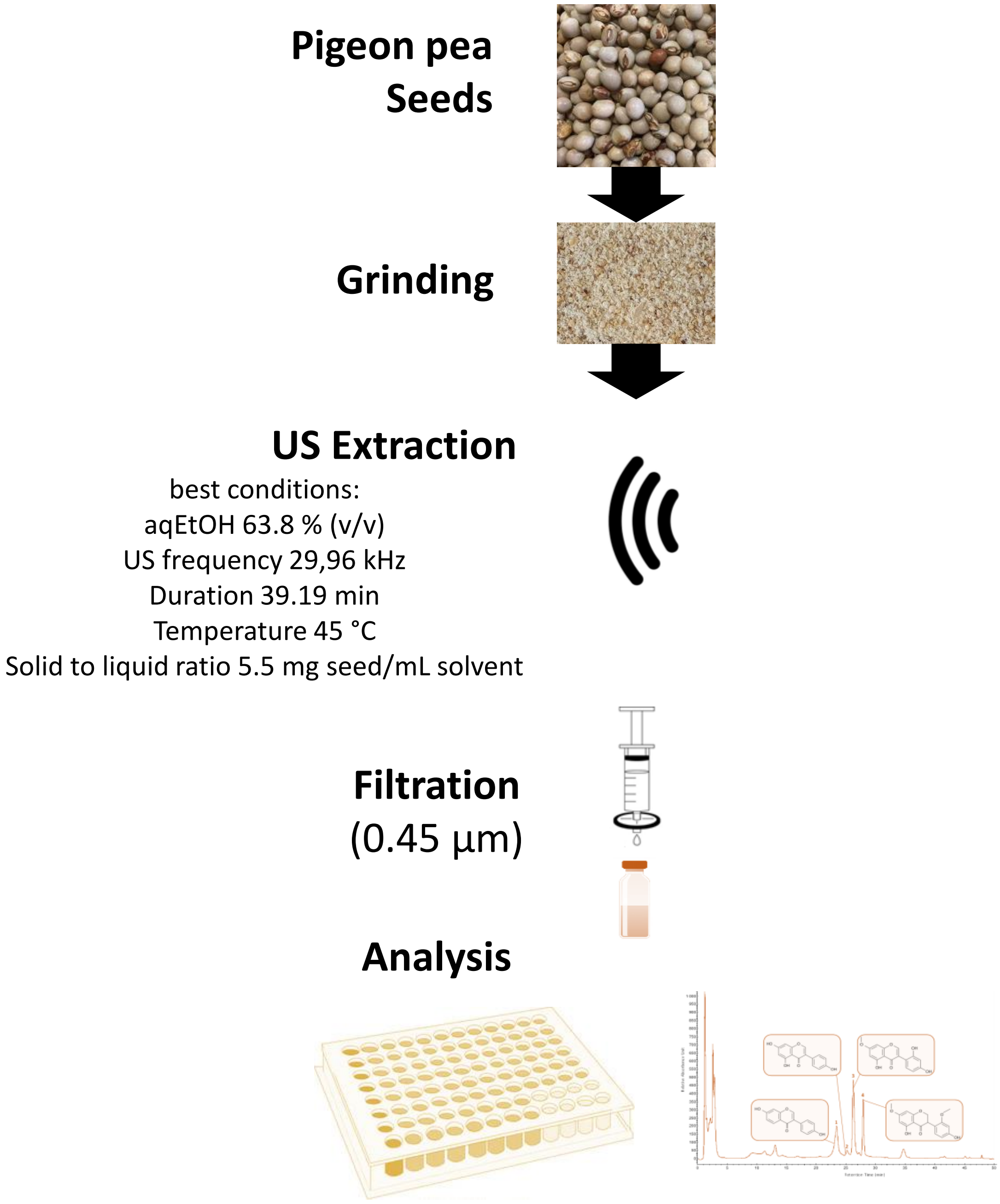

Figure S3. Workflow of the optimized USAE of (iso)flavonoids from pigeon pea seeds.
